# Differentiation of glioblastoma multiforme, metastases and primary central nervous system lymphomas using multiparametric perfusion and diffusion MR imaging of a tumor core and a peritumoral zone—Searching for a practical approach

**DOI:** 10.1371/journal.pone.0191341

**Published:** 2018-01-17

**Authors:** Małgorzata Neska-Matuszewska, Joanna Bladowska, Marek Sąsiadek, Anna Zimny

**Affiliations:** Department of General and Interventional Radiology and Neuroradiology, Wroclaw Medical University, Wroclaw, Poland; University of Portsmouth, UNITED KINGDOM

## Abstract

**Introduction:**

In conventional MR examinations glioblastomas multiforme (GBMs), metastases and primary CNS lymphomas (PCNSLs) may show very similar appearance. The aim of the study was to evaluate usefulness of multiparametric T2*DSC perfusion and diffusion MR imaging in the preoperative differentiation of these tumors.

**Material and methods:**

Seventy four solitary enhancing tumors (27 GBMs, 30 metastases, 17 PCNSLs) were enrolled in the study. Parameters of cerebral blood volume (rCBV), peak height (rPH), percentage of signal recovery (rPSR) and apparent diffusion coefficient (ADC) were assessed from the tumor core and the peritumoral non-enhancing T2-hyperintense zone.

**Results:**

Within the tumor core there were no differences in perfusion and diffusion parameters between GBMs and metastases. Compared to GBMs and metastases, PCNSLs showed significantly lower rCBV and rPH, ADC as well as higher rPSR values. Max rCBV with a cut-off value of 2.18 demonstrated the highest accuracy of 0.98 in differentiating PCNSLs from other tumors. To distinguish GBMs from metastases analysis of the peritumoral zone was performed showing significantly higher rCBV, rPH and lower ADC values in GBMs with the highest accuracy of 0.94 found for max rCBV at a cut-off value of 0.98.

**Conclusions:**

Max rCBV seems to be the most important parameter to differentiate GBMs, metastases and PCNSLs. Analysis of max rCBV within the tumor core enables to distinguish hypoperfused PCNSLs from hyperperfused GBMs and metastases while evaluation of max rCBV within the peritumoral zone is helpful to distinguish GBMs showing peritumoral infiltration from metastases surrounded by pure edema.

## Introduction

Pretreatment characterization and differentiation of malignant brain tumors using MR imaging is still a challenging problem in every day practice. The proper initial diagnosis and subsequently adequate treatment significantly influence patients surveillance but the management can differ substantially, depending on the type of a lesion. The important clinical problem is differentiation between glioblastoma multiforme (GBM), metastases and primary central nervous system lymphomas (PCNSLs) which may show very similar appearance on conventional MR sequences as solitary strongly enhancing brain tumors surrounded by a T2-hyperintense edema. The standard treatment of GBMs and metastases consists of surgical resection, radiotherapy, and chemotherapy while PCNSLs should not undergo a surgical management but only chemotherapy [1–4].

Diffusion (DWI) and perfusion weighted imaging (PWI) are well established advanced MR techniques which allow for more detailed analysis of brain tumors and their in vivo differentiation. Diffusion weighted imaging is a sensitive tool that allows quantifying of physiologic alterations in water diffusion which result from microscopic structural changes not detectable with anatomical MR imaging. Water diffusion can be measured with a parameter of apparent diffusion coefficient (ADC). Diffusivity of water depends primarily on the presence of microscopic structural barriers in tissue such as membranes of cell bodies, axons and myelin sheaths that can alter the random motion of water molecules. Highly cellular tumors show areas of restricted diffusion with low ADC values, thus ADC is regarded as a marker of tumor cellularity [5–7].

Perfusion weighted imaging is a method that brings information on cerebral physiology at the capillary level (microvasculature). Among a few PWI techniques dynamic susceptibility contrast (DSC) MR imaging is the most often used. The method is based on the measurements of the MR signal using T2*-weighted sequence during the first pass of a bolus of a paramagnetic contrast agent. DSC MRI provides maps of cerebral blood volume (CBV) and noninvasive measurements of relative cerebral blood volume (rCBV) which in brain tumors is defined as the ratio between CBV within the tumor and CBV in the white matter of the contralateral hemisphere [8, 9]. rCBV parameter correlates with tumor vascularity and thus is increased in tumors with high rate of pathologic neoangiogenesis, and in gliomas it correlates with the tumor grade [10–15]. The usefulness of other perfusion parameters derived from perfusion curves such as peak height (PH) or percentage of signal recovery (PSR) has also been recently reported with the conclusion that these parameters might be a better criterion than rCBV for tumor differentiation [16].

In the literature there are many articles on using multiparametric MR studies to differentiate brain tumors. They focus mainly on distinguishing high grade from low grade gliomas or high grade gliomas from metastases or meningiomas [17–22]. Majority of them have discussed only the assessment of a tumor core [13,19], less often a peritumoral region has also been evaluated [18, 23]. To our knowledge there are only few articles focusing on differentiation between GBMs, metastases and lymphomas: one study by Mangla et al. showing only the results of T2*DSC perfusion from the tumor core and the peritumoral region [16], study by Rizzo et al. evaluating only the tumor core using both DWI and T2*DSC perfusion [24] and the last study by Zhao et al. in which both MR techniques were used in the assessment of the tumor core and peritumoral edema but PWI was performed using dynamic contrast enhanced (DCE) method not a T2*DSC technique [25].

The aim of our study was to evaluate the diagnostic role of diffusion and T2*DSC perfusion imaging in differentiation of GBMs, metastases and PCNSLs on the basis of evaluation of both the enhancing tumor core and the surrounding T2-hyperintense edema. We also intended to compare different perfusion and diffusion parameters in order to find the parameter with the highest accuracy in distinguishing the three tumor types and try to establish a simple practical approach based on radiological measurements which could be easily incorporated in the clinical practice. Moreover, we divided our subjects into 2 groups—a working set who was used to establish thresholds of different parameters and the second testing group used to validate the initial results.

To our knowledge this is the first publication discussing both DWI and T2*DSC perfusion techniques in the tumoral and peritumoral regions in GBMs, metastases and PCNSLs.

## Material and methods

The study group consisted of 74 solitary enhancing brain tumors which were selected from a cohort of 1210 subjects with CNS tumors evaluated with T2*DSC perfusion and DWI in our institution between January 2010 and July 2017. Our material included 27 patients with biopsy proven GBM (mean age: 61 yrs), 30 with metastases (mean age: 64.5 yrs) and 17 with PCNSL (mean age: 62 yrs). All tumors were located supratentorially and appeared as single strongly enhancing, well delineated lesions surrounded by a T2-hyperintense edema. Sixteen metastases originated from lung cancer, 4 from renal cancer, 2 from intestinal cancer, 5 from breast cancer and 3 were of an unknown origin. All PCNSLs were B-cell lymphomas. The tumors were divided into two groups: working set with known histology (20 GBMs, 20 metastases and 16 lymphomas) used to determine cut off values differentiating different tumor types and a testing set of 18 tumors used to validate the established thresholds.

### Ethics statement

After receiving a written consent all patients underwent MR studies of the brain with contrast enhancement, including diffusion and perfusion sequences. The study was conducted in accordance with the guidelines of the local University Ethics Committee for conducting research involving humans. All procedures were performed with accordance to the Helsinki Human Right consensus and the study was approved by the Commission of Bioethics at Wroclaw Medical University (number of permission: KB-119/2017).

### Data acquisition

All examinations were performed on a 1.5 T MR scanner (Signa Hdx, GE Medical Systems) using a 16-channel HNS (head-neck-spine) coil. Before contrast administration a standard MR examination was carried out including axial T1-weighted images, axial, coronal and sagittal T2-weighted images as well as axial FLAIR images, DWI and post contrast T2*DSC perfusion followed by 3D T1-weighted imaging. During the whole MR examination the patients were instructed to keep their eyes closed. No sedation or anesthesia were used in any of the patients.

#### Perfusion weighted imaging (PWI)

Perfusion examination was performed with a dynamic susceptibility contrast (DSC) method using fast echoplanar T2*-weighted gradient echo sequence with the following parameters: TR = 1.900 ms, TE = 80 ms, FOV = 30 cm, matrix = 192 x 128, slice thickness = 8 mm without spacing, NEX—1.0. Ten seconds after the start of the image acquisition a bolus of a 1.0 mol/l gadobutrol formula (Gadovist, Bayer Health Care, Germany) in a dose of 0.1 ml/kg of a body weight was injected via a 20-gauge catheter placed in the antecubital vein. Contrast material was administered with an automatic injector (Medrad) at a rate of 5 ml/s and was followed by a saline bolus (20 ml at 5 ml/s). The whole perfusion imaging lasted 1 min 26 s in which sets of images from 13 axial slices were obtained before, during and after contrast injection. After PWI a post-contrast 3D T1-weighted sequence was performed using contrast bolus administered earlier for the perfusion examination. No contrast agent was administered before PWI.

#### Diffusion weighted imaging (DWI)

DWI was performed using a transverse single-shot echoplanar diffusion-weighted sequence with the following parameters: TE 89.9 ms, TR 8000 ms, slice thickness– 5 mm, FOV 26 cm, matrix size 128 x 128, NEX—1, diffusion sensitive gradient b = 1000 s/mm^2^ in the three orthogonal directions, scanning time: 42 seconds.

### Image postprocessing

The PWI and DWI images were postprocessed using Functool software (ADW 4.4, GE Medical Systems).

#### Perfusion weighted imaging

**Fig 1:**
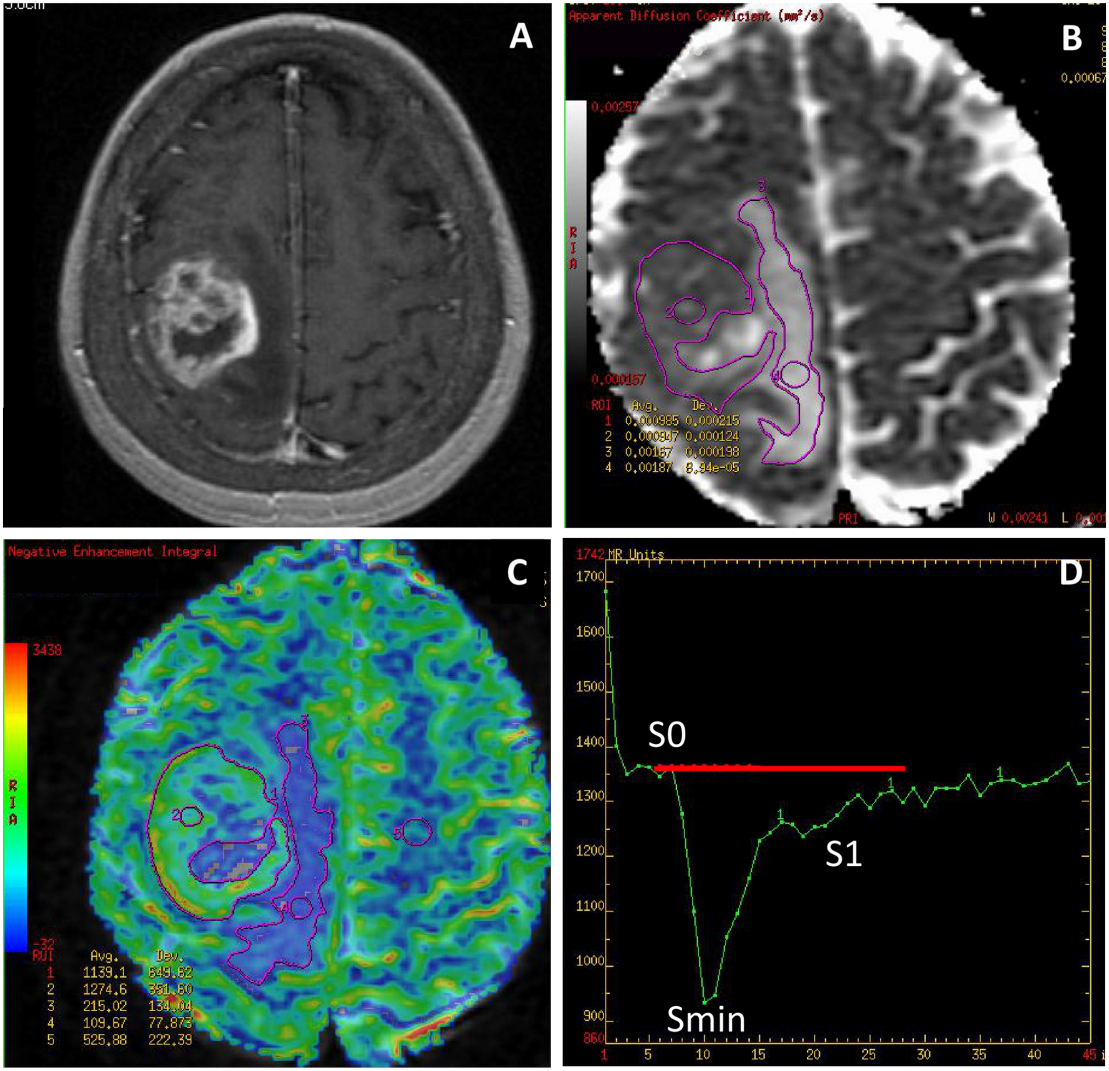
Glioblastoma multiforme (GBM). (A) a post-contrast T1-weighted image, (B) an ADC map, (C) a CBV map, (D) a perfusion signal intensity time curve. The ADC (B) and CBV (C) maps show placement of large irregular freehand ROIs within the tumor core and the peritumoral edema as well as small circular ROIs within the big ROIs and in the contralateral normal white matter used as a reference ROI. The perfusion curve (D) shows hemodynamics of contrast agent during the first pass of a bolus through the brain vasculature with an x-axis reflecting time in seconds and y-axis indicating signal intensity. Red transverse line is a baseline.

The analysis was based on the evaluation of CBV parameters from CBV maps as well as values of peak height (PH) and percentage of signal recovery (PSR) derived from perfusion curves. Measurements of CBV were performed by placing Regions of Interest (ROIs) while PSR and PH values were calculated from the acquired perfusion curves based on formulas: PSR = (S1-Smin) / PH, PH = S0-Smin, where: S0—start of a contrast passage, Smin—maximal drop of magnetic susceptibility, S1—measurement after 24 seconds from Smin (Fig 1). All CBV, PH and PSR values were normalized to the values from the normal appearing white matter of the contralateral hemisphere in order to obtain relative values of all parameters (rCBV, rPH, rPSR) (Fig 1).

Measurements of perfusion parameters were processed within a tumor core and a peritumoral zone. The tumor core was defined as an enhancing part of the tumor on the CBV map fused with the post-contrast T1-weighted image while the peritumoral zone was defined as a T2-hyperintense non-enhancing zone surrounding the tumor core on the CBV map fused with a T2-weighted image.

Measurements of perfusion parameters were obtained for the entire tumor core (mean rCBV, mean rPH, mean rPSR) using large irregular freehand ROIs outlining the enhancing part of a tumor on each slice on the CBV map and subsequently calculating the arithmetical averages from all measured values. Maximal values of these parameters (max rCBV, max rPH, max rPSR) were obtained by placing small ROIs (40–60 mm^2^) over several hot spots within large ROIs on each slice of the CBV map (Fig 1). The highest rCBV value from all ROIs was chosen as the tumoral maximal value. The highest values of rPH and rPSR derived from perfusion curves were accepted as the tumoral maximal values.

T1- and T2-weighted as well as post-contrast T1-weighted images overlaid on CBV maps were used to avoid inclusion of any hemorrhage, necrosis or big vessels within the ROIs.

#### Diffusion weighted imaging

Measurements of ADC for the entire tumor (mean ADC) and measurements of minimum ADC (min ADC) were performed on ADC maps fused with post-contrast T1- weighted images. Similarly to perfusion evaluation, mean ADC values were obtained by manual outlining of the entire enhancing tumor core on each slice avoiding foci of hemorrhage or necrosis and then by calculating the arithmetical averages from all measured ADC values. Min ADC values were measured using small ROIs (40–60 mm^2^) located within the large freehand ROIs. The lowest value from all ROIs was chosen as the tumoral minimum ADC value (Fig 1).

In the hyperperfused tumors such as GBMs and metastases DWI and PWI analysis was also performed in the peritumoral non-enhancing area of T2-hyperintensity by obtaining mean ADC and min ADC values as well as mean rCBV, max rCBV, mean rPH, max rPH, mean rPSR, max rPSR in the manner similar to the measurements within the tumor core. Mean values of diffusion and perfusion parameters were obtained using large irregular freehand ROIs outlining the non-enhancing T2-hyperintense peritumoral zone while max rCBV and min ADC values were calculated using a small ROI method (Fig 1). Mean and max rPH and rPSR values were derived from the perfusion curves acquired for the ROIs placed on the CBV maps.

Methods available at: http://dx.doi.org/10.17504/protocols.io.jjjckkn.

### Statistical analysis

Statistical computations were performed using Statistica PL software package version 4.0, and p <0.05 was set as the significance level.

In the working group differences in perfusion and diffusion parameters among GBMs, metastases and PCNSLs were assessed using ANOVA followed by the post hoc Scheffe test used for group comparisons. In order to assess sensitivity, specificity and accuracy of PWI and DWI parameters in distinguishing PCNSLs from GBMs and metastases as well as GBMs from metastases the receiver-operating characteristic (ROC) analysis was performed. The rate of accuracy was based on the area under the ROC curve. Optimal thresholds for the identification of tumor types were determined on the basis of measurements from the working group. Further, 18 tumors from the testing group underwent radiological evaluation by an experienced neuroradiologist (AZ) blinded to the results of their histology who was supposed to classify the tumors to the different subgroups (GBM, metastasis, PCNSL) using the most powerful imaging parameters and established thresholds. The results of this classification were compared to the results of histology.

## Results

### Measurements from the tumor core of GBMs, metastases and PCNSLs in the working group

**Fig 2:**
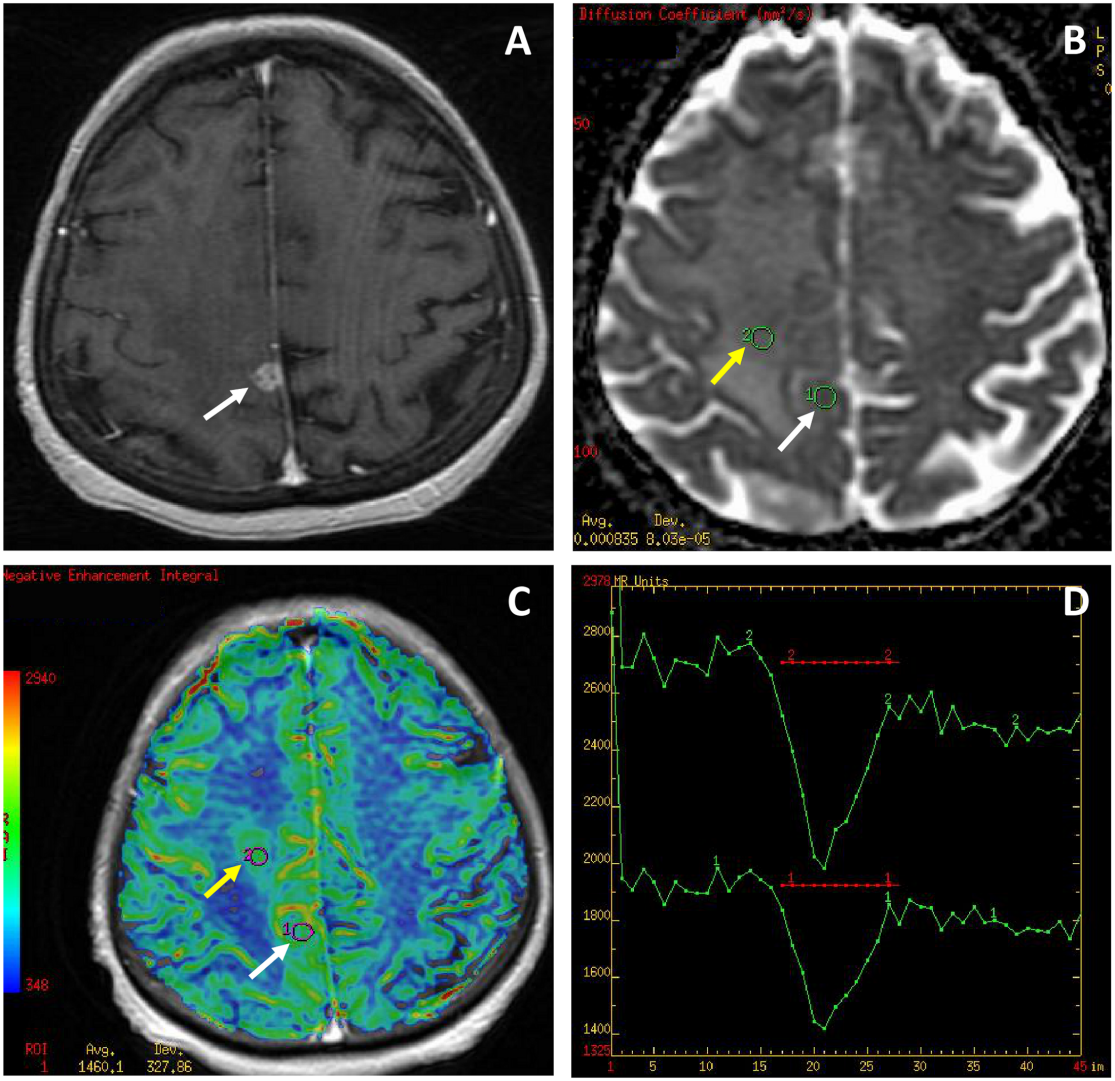
Glioblastoma multiforme (GBM). (A) a post-contrast T1-weighted image, (B) an ADC map, (C) a CBV map, (D) perfusion signal intensity time curves. The tumor appears as a small enhancing lesion (white arrows) with a large non-enhancing peritumoral zone mimicking a metastasis. The ADC map (B) shows minimal ADC values similar to normal white matter (0.83 x 10^−3^ mm^2^/s within the tumor core and 0.78 x 10^−3^ mm^2^/s within the peritumoral region of infiltration). The CBV map (C) shows the hyperperfused tumor core (white arrow, max rCBV = 2.9) and a large area of increased perfusion (yellow arrow, max rCBV = 2.25) within the peritumoral zone indicating neoplastic infiltration which is a feature differentiating GBM from a metastasis surrounded exclusively by a pure vasogenic edema. The perfusion curves (D) present only partial return to the baseline (red transverse line) in both the tumor core (lower curve) and the area of neoplastic infiltration (upper curve).

**Fig 3:**
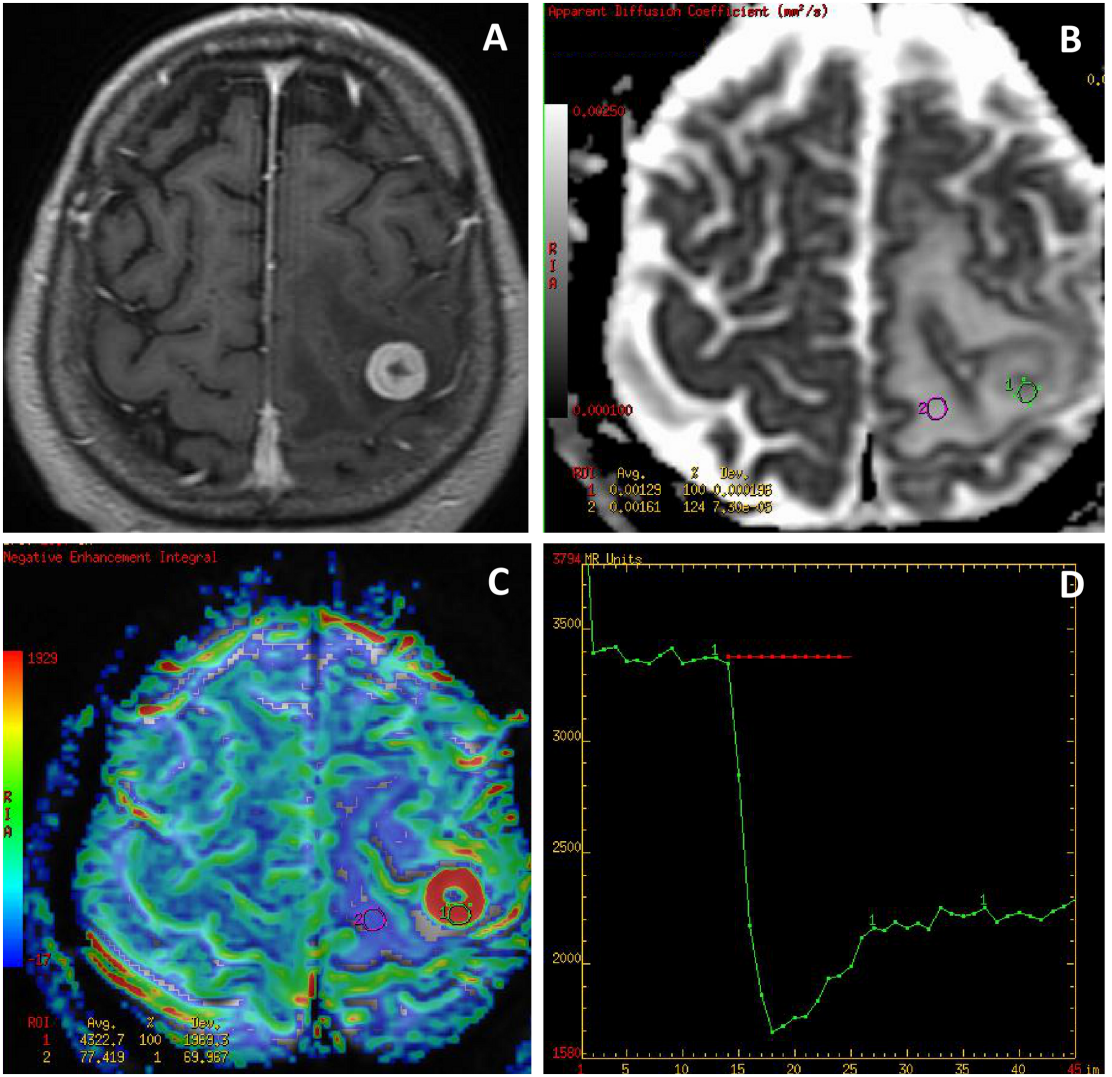
A solitary metastasis. (A) a post-contrast T1-weighted image, (B) an ADC map, (C) a CBV map, (D) a perfusion signal intensity time curve. The ADC map (B) shows facilitated diffusion in both the tumor core (min ADC = 1.0 x 10^−3^ mm^2^/s) and in the peritumoral zone (min ADC = 1.32 x 10^−3^ mm^2^/s). The CBV map (C) shows a highly perfused tumor (max rCBV = 17.9) and no hyperperfusion within the peritumoral zone (max rCBV = 0.78) typical for pure vasogenic edema. The perfusion curve (D) presents partial return to the baseline (red transverse line).

There were no significant differences between GBMs and metastases in the mean values of all evaluated perfusion and diffusion parameters (Table 1) (Figs 2 and 3).

**Table 1:** The values of perfusion and diffusion parameters from the tumor core in GBMs, metastases and PCNSLs with the analysis of variance and multiple post-hoc comparisons among the patient subgroups.

Parameters evaluated (MR technique)	Mean value ± SD (range)			ANOVA p values	Scheffe test, p values		
	GBM	META	PCNSL		GBM vs META	GBM vs PCNSL	META vs PCNSL
mean rCBV (PWI)	3.10±1.50 (1.11–6.89)	4.49±3.94 (1.80–14.88)	0.80±0.35 (0.31–1.41)	**0.0001**	0.23	**0.032**	**0.0001**
max rCBV (PWI)	7.65±4.75 (2.09–19.30)	7.53±5.38 (2.50–18.76)	1.32±0.58 (0.44–2.18)	**0.0001**	0.99	**0.0001**	**0.0001**
mean rPH (PWI)	2.79±1.29 (1.26–6.45)	3.15±2.01 (1.54–8.88)	1.16±0.40 (0.74–2.40)	**0.0001**	0.73	**0.006**	**0.001**
max rPH (PWI)	5.55±2.93 (1.27–12.39)	5.23±2.90 (2.57–12.16)	1.79±0.73 (0.86–3.69)	**0.0001**	0.91	**0.0001**	**0.001**
mean rPSR (PWI)	1.17±0.28 (0.79–2.08)	1.19±0.64 (0.50–3.50)	1.92±0.75 (1.22–3.78)	**0.0001**	0.99	**0.001**	**0.002**
max rPSR (PWI)	1.32±0.40 (0.87–2.73)	1.30±0.65 (0.59–3.50)	2.29±0.19 (1.24–5.83)	**0.0001**	0.99	**0.002**	**0.002**
mean ADC* (DWI)	1.02±0.17 (0.69–1.47)	1.03±0.19 (0.73–1.34)	0.73±0.13 (0.57–1.04)	**0.0001**	0.98	**0.0001**	**0.0001**
min ADC* (DWI)	0.72±0.15 (0.47–1.13)	0.76±0.20 (0.46–1.18)	0.57±0.12 (0.40–0.82)	**0.002**	0.73	**0.024**	**0.003**

*ADC values should be multiplied by 10^−3^ and expressed in units of mm^2^/s.

ADC, apparent diffusion coefficient; DWI, diffusion weighted imaging; GBM, glioblastoma multiforme; META, metastasis; max, maximum; min, minimum; PCNSL, primary CNS lymphoma; PWI, perfusion weighted imaging; rCBV, relative cerebral blood volume; rPH, relative peak height; rPSR, relative percentage of signal recovery.

**Fig 4:**
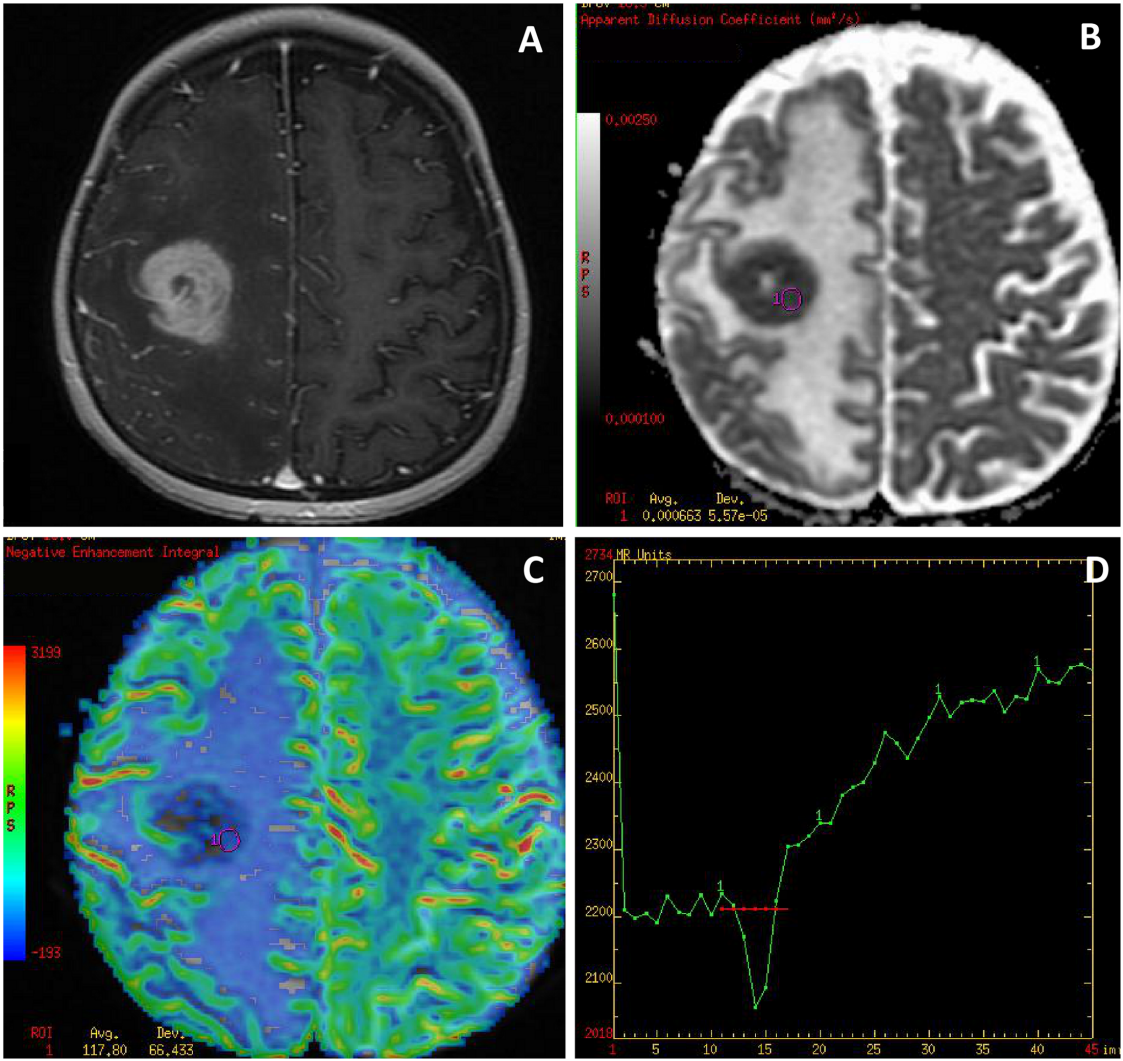
Primary central nervous system lymphoma (PCNSL). (A) a post-contrast T1-weighted image, (B) an ADC map, (C) a CBV map, (D) a perfusion signal intensity time curve. The tumor appears as an enhancing solitary lesion with a large peritumoral edema mimicking a metastasis. The ADC map (B) shows restricted diffusion within the tumor core (min ADC = 0.51 x 10^−3^ mm^2^/s). The CBV map (C) shows the hypoperfused tumor core (max rCBV = 0.79) with the perfusion curve (D) returning above the baseline (red transverse line) which are typical perfusion characteristics of PCNSL.

Compared to metastases and GBMs, PCNSLs showed significant differences in all evaluated parameters such as significantly lower values of mean rCBV, max rCBV, mean rPH and max rPH as well as significantly higher mean rPSR and max rPSR values. Diffusion parameters of PCNSLs were significantly lower compared to GBMs and metastases (Table 1) (Fig 4).

Comparing PCNSLs and metastases, there was no overlap in the values of mean rCBV and max rCBV (mean rCBV range for PCNSLs 0.31–1.41 and for metastases 1.80–14.88, max rCBV range for PCNSLs 0.44–2.18 and for metastases 2.50–18.76) while in one case of GBM (5% of all GBMs) both mean rCBV (1.11) and max rCBV (2.09) reached the low values of PCNSLs. There was also a certain overlap in the values of rPSR, rPH and ADC between PCNSLs and metastases or GBMs (Table 1).

**Fig 5:**
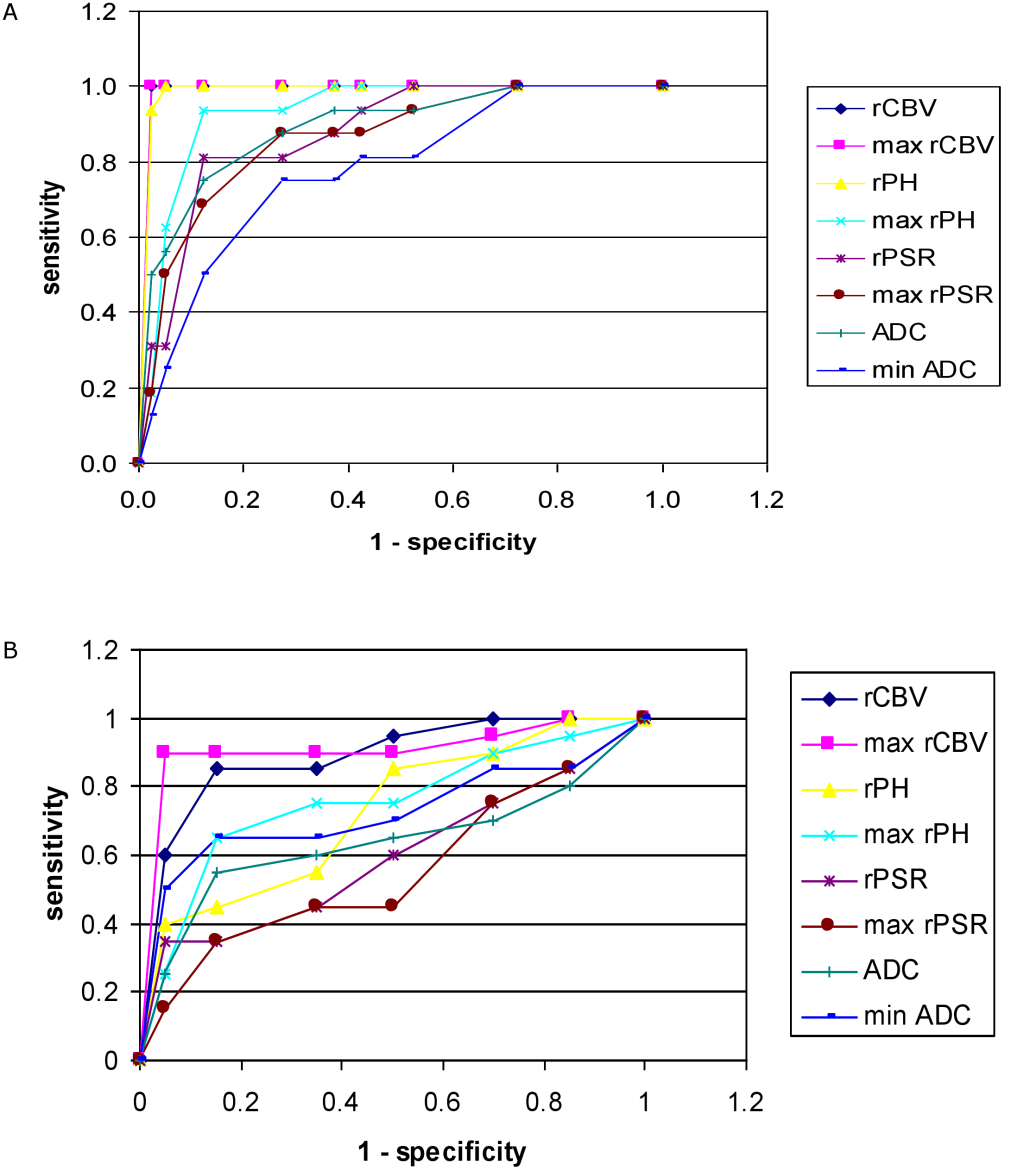
Receiver operating characteristic (ROC) curve. ROC analysis for comparisons of perfusion and diffusion parameters from the tumor core (A) which are used for differentiation of PCNSLs from GBMs and metastases as well as from the peritumoral zone (B) used for differentiation of GBMs from metastases. Both graphs A and B show the highest accuracy (the largest area under the curve) for the values of max rCBV.

From all parameters differentiating PCNSLs from GBMs and metastases, the highest accuracy was found for max rCBV and mean rCBV with cut-off values of 2.18 and 1.41, respectively, followed by mean rPH, max rPH, mean ADC, mean rPSR and max rPSR (Table 2) (Fig 5).

**Table 2:** The results of ROC analysis for differentiation of PCNSLs from GBMs and metastases as well as GBMs from metastases.

**PCNSLs vs (GBMs + metastases)**				
**Parameter**	**Accuracy**	**Cut-off value**	**Specificity**	**Sensitivity**
mean rCBV	0.984	< 1.41	0.975	1.0
max rCBV	0.985	< 2.18	0.975	1.0
mean rPH	0.969	< 1.49	0.975	0.938
max rPH	0.938	< 3.01	0.875	0.938
mean rPSR	0.860	> 1.39	0.875	0.813
max rPSR	0.851	> 1.65	0.725	0.875
mean ADC*	0.903	< 0.81*	0.850	0.875
min ADC*	0.777	< 0.56*	0.850	0.625
**GBMs vs metastases**				
**Parameter**	**Accuracy**	**Cut-off value**	**Specificity**	**Sensitivity**
mean rCBV	0.911	> 0.6	0.85	0.85
max rCBV	0.940	> 0.98	1.0	0.9
mean rPH	0.699	> 0.94	0.5	0.85
max rPH	0.763	> 2.05	0.85	0.65
mean rPSR	0.573	< 0.99	0.85	0.35
max rPSR	0.538	< 1.13	0.85	0.35
mean ADC*	0.628	< 1.49*	0.85	0.55
min ADC*	0.721	< 1.1*	0.9	0.65

*ADC values should be multiplied by 10^−3^ and expressed in units of mm^2^/s.

ADC, apparent diffusion coefficient; GBM, glioblastoma multiforme; max, maximum; min, minimum; PCNSL, primary CNS lymphoma; rCBV, relative cerebral blood volume; rPH, relative peak height; rPSR, relative percentage of signal recovery.

### Measurements of the T2-hyperintense peritumoral zone in GBMs and metastases in the working group

Compared to metastases, GBMs showed significantly higher values of mean rCBV, max rCBV, mean rPH, max rPH and lower values of min ADC (Figs 2 and 3). No significant differences in the values of mean rPSR and max rPSR as well as mean ADC were observed between GBMs and metastases (Table 3).

**Table 3:** The values of perfusion and diffusion parameters from the peritumoral zone in GBMs and metastases with the comparisons between the patient subgroups.

Parameters evaluated (MR technique)	Mean value ± SD (range)		p values
	GBM	META	
mean rCBV (PWI)	1.05±0.39 (0.56–1.89)	0.55±0.13 (0.35–0.77)	**0.0001**
max rCBV (PWI)	2.07±0.75 (0.70–3.75)	0.78±0.15 (0.53–0.98)	**0.0001**
mean rPH (PWI)	1.27±0.38 (0.59–1.96)	1.01±0.36 (0.43–1.77)	**0.035**
max rPH (PWI)	2.22±0.84 (0.76–3.84)	1.53±0.56 (0.66–2.71)	**0.004**
mean rPSR (PWI)	1.15±0.25 (0.76–1.62)	1.21±0.20 (0.89–1.58)	0.414
max rPSR (PWI)	1.40±0.35 (0.90–2.19)	1.44±0.36 (1.07–2.58)	0.740
mean ADC* (DWI)	1.47±0.27 (0.99–1.90)	1.58±0.15 (1.16–1.79)	0.123
min ADC* (DWI)	1.07±0.32 (0.60–1.62)	1.31±0.13 (0.99–1.48)	**0.005**

*ADC values should be multiplied by 10^−3^ and expressed in units of mm^2^/s.

ADC, apparent diffusion coefficient; DWI, diffusion weighted imaging; GBM, glioblastoma multiforme; META, metastasis; max, maximum; min, minimum; PWI, perfusion weighted imaging; rCBV, relative cerebral blood volume; rPH, relative peak height; rPSR, relative percentage of signal recovery.

Max rCBV and mean rCBV were the parameters which revealed the highest accuracy in distinguishing GBMs from metastases (with cut-off values above 0.98 and 0.6, respectively) though there was an overlap in the values of both mean rCBV and max rCBV between the two tumor groups (Tables 2 and 3) (Fig 5). Two cases of GBMs out of 20 (10%) similarly to metastases did not show areas of high max rCBV values in the peritumoral zone and 7 patients out of 20 GBMs (35%) showed the same results of mean rCBV as metastases (Table 3).

All detailed diffusion and perfusion measurements are available in the “S1 Table” in the supporting information file.

### Results of the testing set

Based on the results of the working group which revealed that max rCBV and mean rCBV values show the highest accuracy in differentiation between GBMs, metastases and PCNSLs, only these two parameters were used for evaluation of the testing set. In the first step, the results from the tumor core of the testing set were assessed showing mean values of max rCBV = 6.8 (range: 1.42–16.75) and mean values of mean rCBV = 3.72 (range: 0.77–8.66) (Table 4). The max rCBV cut-off value of 2.18 was used to discriminate between hypoperfused PCNSLs and hyperperfused GBMs and metastases revealing only one tumor with max rCBV values lower than 2.18 thus classified as PCNSL. The other tumors with max rCBV values higher than 2.18 underwent the second part of evaluation which was assessment of the non-enhancing peritumoral zone showing the following results: mean values of max rCBV = 1.28 (range: 0.57–2.81) and mean values of mean rCBV = 0.72 (range: 0.38–1.28). Using the cut-off value of max rCBV as 0.98, 11 out of 17 hyperperfused tumors were classified as metastases (with max rCBV below 0.98) while 6 were classified as GBMs (with max rCBV above 0.98).

**Table 4:** Perfusion results of the testing group with the comparison of the suggested and biopsy proven diagnosis.

	Tumor core		Peritumoral zone		Suggested diagnosis	Biopsy results
	mean rCBV	max rCBV	mean rCBV	max rCBV		
T1	0.77	1.42			PCNSL	PCNSL
T2	2.15	3.63	0.48	0.74	metastasis	metastasis
T3	6.42	11.74	0.45	0.8	metastasis	metastasis
T4	4.33	9.1	0.5	0.79	metastasis	metastasis
T5	2.92	4.82	0.49	0.78	metastasis	metastasis
T6	2.86	4.31	0.38	0.57	metastasis	metastasis
T7	2.75	5.09	0.52	0.75	metastasis	metastasis
T8	8.66	11.78	0.58	0.83	metastasis	metastasis
T9	4.25	7.92	0.78	0.9	metastasis	metastasis
T10	2.33	4.9	0.54	0.73	metastasis	metastasis
T11	8.43	11.89	0.68	0.93	metastasis	metastasis
T12*	3.4	5.02	0.73	0.8*	metastasis	GBM
T13	1.72	3.53	1.01	2.48	GBM	GBM
T14	2.77	7.01	0.75	1.84	GBM	GBM
T15	2.57	5.76	0.67	1.4	GBM	GBM
T16	1.57	2.73	1.13	2.03	GBM	GBM
T17	2.68	5.15	1.28	2.81	GBM	GBM
T18	6.44	16.75	1.27	2.6	GBM	GBM

* misdiagnosed tumor; GBM, glioblastoma multiforme; max, maximum; PCNSL, primary CNS lymphoma; rCBV, relative cerebral blood volume; T, tumor.

All metastases and one PCNSL were correctly diagnosed, while one case of GBM with low max rCBV values in the peritumoral zone was misinterpreted as a metastasis (Table 4). Overall 17 out of 18 tumors were correctly diagnosed based on perfusion parameters reaching the accuracy of 0.94.

## Discussion

Our study illustrates the utility of DWI and PWI using T2*DSC technique for differentiation of GBMs, metastases and PCNSLs, which is still very challenging in standard MR imaging, especially when these tumors present as enhancing solitary brain lesions surrounded by a T2-hyperintense edema. In our study we focused on both the tumor core and the brain tissue surrounding the tumor since they are considered two equally important sources of information necessary for tumor identification.

The analysis of the tumor core did not show any significant differences between GBMs and metastases regarding diffusion and perfusion parameters. These two types of tumors appeared as highly perfused lesions with high rCBV and rPH values with partial return of the perfusion curve to the baseline (low PSR values), and normal or facilitated diffusion reflected by medium to high ADC values (Figs 2 and 3). These findings are well known in the literature and have been reported before [12, 13, 18–20, 24]. Several reports have demonstrated that GBMs and metastases may reach very high max rCBV values of 3.0 or even 10.0 and max rCBV of 1.75 has been set as the threshold value differentiating low grade from high grade gliomas [17]. There are also several studies reporting that tumoral rCBV values may not be sufficient for discriminating metastatic tumors from high-grade gliomas [20, 22], indicating that other parameters should also be used such as PSR but the results are contradictory [16]. The reports on usefulness of ADC values in differentiation of GBMs and metastases are also contradictory, some authors suggest that the tumoral ADC values are not useful for discriminating metastatic tumors from high-grade gliomas [6, 7, 13] while some show opposite results [22].

In our study PCNSLs showed characteristic perfusion and diffusion patterns, which were very different from GBMs and metastases, such as hypoperfusion within the tumor core with low rCBV and rPH values, a perfusion curve with the return over the baseline reflected by high rPSR values as well as restricted diffusion with low ADC values (Fig 4). Similar findings were reported before in several diffusion and perfusion studies on lymphomas [16, 26–29].

Different rCBV and ADC values of PCNSLs compared to GBMs and metastases may be explained by their histology [3]. In contrary to GBMs and metastases lymphomas have highly concentrated large cells and smaller extravascular space which are dominant causes of typically low mean ADC values reflected by homogeneously restricted diffusion within the entire tumor core. Hypoperfusion in lymphomas can be explained by hypovascularization and absence of neoangiogenesis [16]. The exact explanation of the signal intensity time curve returning above the baseline level (high rPSR values) is difficult and not fully understood. It is probably due to gadolinium extravasation into the interstitial space and complex T1 and T2 effects which can alter the shape of the perfusion curve. Other physiologic factors such as blood flow, blood volume, vascular permeability, and leakage space or the interplay between these factors may also lead to high PSR [16].

However in some studies lymphomas were reported to show higher values of rCBV, but this requires different perfusion technique either with a preloading bolus of contrast or correction of rCBV during postprocessing [9, 30]. In our opinion this approach is not very useful in clinical practice since low rCBV values in PCNSLs are very important features differentiating them from GBMs and metastases. When performing perfusion with a preloading bolus or correction of rCBV in the postprocessing stage, PCNSLs become highly perfused tumors with increased rCBV values similar to GBMs making differentiation between these two entities very difficult [30].

Our findings regarding utility of perfusion parameters are in contradiction with the results of Mangla et al. who found parameters of rPSR to have higher accuracy than rCBV in differentiating lymphomas from GBMs and metastases [16]. In our study max and mean rPSR values were also capable of differentiating PCNSLs from other tumors but with less accuracy (0.86) than max rCBV and mean rCBV (0.98) and even less accuracy than max rPH and mean rPH (0.93 and 0.96, respectively) as well as mean ADC values (0.9). In our opinion from the practical point of view analysis of perfusion examinations may be narrowed to the visual assessment of perfusion maps to evaluate mean rCBV of the entire tumor and then searching for hot spots within the tumor core and simple analysis of max rCBV which is not a time consuming procedure compared to the parametric analysis of perfusion curves. Evaluation of max rCBV may also be followed by assessment of mean ADC values as this parameter showed higher accuracy than PSR. Assessment of both max rCBV and ADC values is easily accessible on workstations of all vendors. The visual evaluation of the shape of perfusion curves is also not a time consuming procedure and overshooting of the curve which is typical for lymphomas may be easily detected. On the other hand, the detailed evaluation of the parameters derived from perfusion curves such as rPH and rPSR is more complicated and requires more time. In our opinion evaluation of these parameters is not necessary and in the everyday practice may be omitted since it does not have high accuracy in distinguishing lymphomas from GBMs and metastases.

Since metastases and GBMs tend to show very similar perfusion and diffusion patterns due to similar rate of neovascularization and cellularity within the enhancing parts of these tumors, these measurements cannot be used to accurately differentiate these tumors. The next step in our study was to evaluate a non-enhancing peritumoral region in order to distinguish GBMs from metastases. Highly aggressive nature of GBMs is associated with their infiltrative growth in the peritumoral area exceeding the limits of an enhancing tumor core, while metastases arise within the brain parenchyma and usually grow by expansion, displacing the surrounding brain tissue, which is reflected by purely vasogenic peritumoral edema [10]. Peritumoral zones in the regions of GBM infiltration have been already evaluated in the literature using DWI or PWI showing lower ADC values [18, 23, 31] and increased rCBV values [22, 32]. In our study we also found significantly increased values of rCBV and rPH as well as significantly decreased min ADC values reflecting neoplastic infiltration in the peritumoral zone surrounding GBM.

In our study of the peritumoral zone the parameters with the highest accuracy in differentiating GBMs from metastases were max rCBV followed by mean rCBV reaching the values of 0.94 and 0.91, respectively. Since max rCBV reached the highest accuracy in distinguishing GBMs from metastases we suggest to focus only on the measurements of this parameter in the clinical practice which is an easy and fast method of assessment of perfusion maps by searching for the so called hot spots outside the tumor core.

Summarizing our results from the tumor core and peritumoral zone the most important parameter differentiating GBMs, metastases and lymphomas is max rCBV. This finding is in strong opposition to the results of Mangla et al. [16] who performed very similar DSC perfusion study trying to compare the same three types of tumors on the basis of several perfusion parameters derived from a tumor core and a peritumoral zone using the same MR machine (1.5 GE scanner). In contradiction to our work, Mangla et al. did not evaluate the diffusion parameters and reported rPSR to be the most sensitive and specific feature in differentiation lymphomas from GBMs and metastases. In our study the rPSR ratio was helpful in differentiating lymphoma from GBM and metastases but did not differentiate GBMs from metastases and its accuracy was much lower compared to other perfusion and diffusion parameters.

We have to remember that still even though max rCBV shows the highest sensitivity and specificity there is a slight overlap in the values between the evaluated tumor types. When evaluating the tumor core, 1 case of GBM showed low max rCBV similarly to lymphomas and when assessing the peritumoral zone in 2 cases of GBM tumors we did not find foci of neoplastic infiltration reflected by increased max rCBV. To reach the highest accuracy we propose to use the cut-off values of max rCBV = 2.18 within the tumor core and max rCBV = 0.98 within the peritumoral zone. Blasel et al. also showed that using the cut-off value of rCBV = 1.0 within the peritumoral zone for discriminating metastases from GBMs yielded a sensitivity of 96% and specificity of 64% [32]. To validate our initial results and established thresholds we also performed second analysis of a new cohort of 18 patients with solitary brain tumors. The second analysis revealed very similar results to the first one and proved that the established thresholds of max rCBV are useful parameters in the differentiation between GBMs, metastases and PCNSLs and may be very easily incorporated in the clinical everyday routine. On the basis of the evaluation of the max rCBV from the tumor core and the peritumoral zone 16 out of 17 tumors were correctly diagnosed, and the tumor who wasmisinterpreted was GBM similarly to the results of the working group.

### Practical approach to the evaluation of brain tumors

We propose a simple two-step approach to evaluation of enhancing brain tumors based only on the assessment of max rCBV values within the tumor core and then if necessary also within a non-enhancing peritumoral zone. First step is the evaluation of the tumor core and dividing brain tumors into two major groups hyperperfused (max rCBV above 2.18) indicating GBMs or metastasis and hypoperfused (max rCBV below 2.18) suggesting PCNSLs. The second step important only in the case of hyperperfused tumors is an assessment of the peritumoral non-enhancing tissue in search for foci of tumor infiltration (max rCBV above 0.98) which may be found in the majority of GBM lesions but not in metastases. Moreover, we would like to emphasize that assessment of max rCBV using the small ROI method is an easy, practical and not time consuming procedure which can be easily incorporated in the everyday clinical practice. The small ROI method does not require long training and was proved to demonstrate very good interobserver and intraobserver reproducibility [33].

## Conclusions

In our opinion analysis of diffusion and perfusion parameters should be used to differentiate GBMs, metastases and PCNSLs, which may look similarly on standard MR examinations as strongly enhancing focal brain lesions surrounded by edema. In the clinical practice we recommend a two-step approach based on evaluation of the most important parameter which is max rCBV first within the tumor core to distinguish hyperperfused (GBMs and metastases) from hypoperfused (PCNSLs) tumors, and secondly within the peritumoral zone of the hyperperfused tumors to search for neoplastic infiltration typical for GBM or pure vasogenic edema characteristic for metastases. Evaluation of other diffusion and perfusion parameters may bring additional helpful information. DWI and PWI which are techniques easy to perform and fast to postprocess should be incorporated in the MR protocol of all intracranial tumors.

## Supporting information

S1 Table All perfusion and diffusion measurements from metastases, GBMs and PCNSLs in the working group. (XLSX)
